# Editorial: Diagnosis and treatment of vulvar cancer

**DOI:** 10.3389/fonc.2025.1569255

**Published:** 2025-03-04

**Authors:** Violante Di Donato, Tullio Golia D’Augè, Giorgio Bogani, Andrea Giannini

**Affiliations:** ^1^ Department of Maternal and Child Health and Urological Sciences, Sapienza University of Rome, Policlinico Umberto I, Rome, Italy; ^2^ Gynecologic Oncologic Unit, Fondazione IRCCS Istituto Nazionale dei Tumori, Milano, Italy; ^3^ Unit of Gynecology, Department of Surgical and Medical Sciences and Translational Medicine, Sant’Andrea Hospital, Sapienza University of Rome, Rome, Italy

**Keywords:** vulvar cancer, HPV, radiotherapy, biomarkers, molecular profiling, gynecologic oncology, early detection, personalized medicine

Vulvar cancer, although a rare gynecological cancer, presents noteworthy challenges in diagnosis, treatment and outcomes (1, 2). It requires a highly tailored approach to manage the medical and psychosocial aspects, carefully considering functionality and aesthetic impact (3–5). While the incidence is lower compared to other gynecological cancers, vulvar malignancies share similar complexities surrounding early detection, staging, and therapeutic interventions (5–7). Indeed, in Italy, the incidence of vulvar squamous cell carcinoma has been rising in women under 50, with an estimated annual increase of +1.20% from the 90s (1). The main aim of this Research Topic was to explore advancements in the diagnosis, treatment, and management of vulvar cancer, with a particular focus on improving therapeutic approaches, diagnostic strategies and patient quality of life. The studies presented in this Research Topic highlight significant developments in understanding the molecular and clinical aspects of vulvar cancer, addressing challenges in treatment and the need for innovative management approaches. A total of six high-quality papers were published on this Research Topic: two systematic reviews, three original research articles and one review article. Together, these studies underline the complexity of vulvar cancer management, from early detection and staging to personalized treatment options, highlighting the need for improvements in strategies and outcomes.

A study by Emagneneh et al. highlights several persistent issues in vulvar cancer management. One of the major global challenges discussed is the lower survival rates for cervical cancer in regions such as Sub-Saharan Africa, where access to preventive screening, early detection, and treatment remains limited. The systematic review reveals that survival rates can be less than 35%, underscoring the disparities in healthcare access. Though vulvar and cervical cancers have distinct etiologies, this study underscores the broader issue of healthcare infrastructure and the need for international collaboration to improve prevention, diagnosis, and treatment access. Efforts to improve HPV vaccination, screening, and early intervention strategies can similarly benefit vulvar cancer care, especially in underserved areas.

The Research Topic also investigates the molecular subtyping of gynecologic cancers, with a study by Yin et al. examining its correlation with clinicopathological features and prognosis. Although the molecular characterization of vulvar cancer is not as advanced, the findings from this research highlight the potential for similar methods to be applied to vulvar malignancies. Identifying specific molecular markers, such as those linked to HPV status, could consent to more precise patient stratification and guide treatment decisions, providing a clearer route toward targeted therapies.

In the context of vulvar pathology, Seol et al. present a retrospective cohort study of Extramammary Paget’s Disease (EMPD) of the vulva, a rare condition often occurring in postmenopausal women. This study explores the role of radiotherapy in preserving vulvar function and aesthetics and maintaining survival outcomes. Although the study sample was limited, the results suggest that radiotherapy may be a feasible option to preserve vulvar tissue without compromising overall survival, offering a less radical treatment approach that could become standard in cases of extensive EMPD.

A notable study published by Li et al. investigates the role of HPV in vulvar squamous cell carcinoma (SCC), demonstrating that HPV-associated tumors have a higher sensitivity to radiotherapy. The meta-analysis shows that patients with HPV-related vulvar cancer have better local control, overall survival and reduced recurrence rates when treated with radiotherapy, according to cervical and oropharyngeal cancers, where HPV status plays a critical role in determining prognosis and therapeutic responsiveness. These findings suggest that HPV biomarkers could be integrated into risk stratification models for vulvar cancer, hypothetically guiding the use of radiotherapy and other treatment modalities.

Moreover, the research by Yan et al. includes studies exploring the use of advanced imaging techniques in gynecologic oncology. The article introduces a radiomics-based nomogram developed to predict lymph node metastasis in endometrial cancer, incorporating MRI features. This approach could be adapted to vulvar cancer for precise staging and personalized treatment, particularly in assessing the nodal status, crucial for determining the appropriate surgical and adjuvant treatment.

Finally, a study by Huang et al. examining biomarkers in advanced ovarian cancer presents the potential for testing several biomarkers to guide neoadjuvant chemotherapy decisions. Although ovarian and vulvar cancers are biologically distinct, the concept of using biomarkers to personalize treatment regimens is highly relevant even to vulvar cancer management. Identifying predictive biomarkers could enable more effective use of chemotherapy and radiation therapy, improving patient outcomes, selecting the most appropriate treatment strategies.

In summary, this Research Topic emphasizes the importance of a multidisciplinary approach to vulvar cancer treatment that integrates molecular profiling, advanced imaging, biomarker-driven therapies and individualized care procedures. These advancements have the potential to drastically improve outcomes for vulvar cancer patients, principally through early detection, personalized therapies and enhanced patient care (1, 3). However, challenges such as healthcare disparities and the need for more accessible treatments persist and must be addressed worldwide.

This Research Topic encourages collaboration and further studies to enhance the management of vulvar cancer, to make future interventions more effective and accessible. By learning from other gynecologic cancers and advancing research on vulvar cancer, we hope to improve survival rates and patient’s quality of life worldwide.
